# *In vitro* Evaluation of *Terminalia arjuna* on Calcium Phosphate and Calcium Oxalate Crystallization

**DOI:** 10.4103/0250-474X.70480

**Published:** 2010

**Authors:** A. Chaudhary, S. K. Singla, C. Tandon

**Affiliations:** Department of Biotechnology and Bioinformatics, Jaypee University of Information Technology, Waknaghat, Solan-173 215, India; 1 Department of Biochemistry, Panjab University, Chandigarh-160 014, India

**Keywords:** Calcium phosphate, calcium oxalate, saponin, *Terminalia arjuna*, TLC, urolithiasis

## Abstract

Urinary stones are one of the oldest and the most common afflictions in humans. This disease has tormented humans since the earliest records of civilization. Ten percent of men and 3 % of women have a stone during their adult lives. Calcium containing stones are the most common comprising about 75 % of all urinary calculi, which may be in the form of pure calcium oxalate (50 %) or calcium phosphate (5 %) or a mixture of both (45 %). A number of plants have been mentioned in the Indian ayurvedic system, which plays a vital role in the inhibition of kidney stones. In the present study, the inhibitory potency of crude extracts or fractions of successive solvent extractions of *Terminalia arjuna* bark was evaluated on various stages of formation of calcium phosphate and on the growth of calcium oxalate monohydrate crystals *in vitro*. Results obtained indicated that *Terminalia arjuna* bark has the potential to inhibit the formation of both calcium phosphate and calcium oxalate crystals *in vitro*. Butanol fraction of *Terminalia arjuna* extract was the most effective in inhibiting formation of calcium phosphate and calcium oxalate crystals *in vitro*.

A number of people suffer from problems due to urinary stones (calculi). As many as 10.0% of men and 3.0% of women have a stone during their adult lives[1]. Areas of high incidence of urinary calculi, include the British Isles, Scandinavian countries, northern Australia, central Europe, northern India, Pakistan and Mediterranean Countries[2]. Saurashtra region of Gujarat has higher prevalence of urinary stones. According to an estimate every year, 6 00 000 Americans suffer from urinary stones[3]. In India, 12% of the population is expected to have urinary stones, out of which 50% may end up with loss of kidneys or renal damage. Also, nearly 15% of the population of northern India suffers from kidney stones[3]. Calcium stones are most common, comprising 75% of all urinary calculi[4]. However, development of modern techniques such as extracorporeal short wave lithotripsy (ESWL)[5] and percutaneous nephrolithotomy (PCNL) have revolutionized surgical management of the problem, yet not much progress has been made towards the medical management of kidney stone problem. Patients may experience discomfort, soreness or pain at the treatment site. A prescription for pain relieving medication or extra strength acetaminophen is recommended. Some patients also show discomfort or pain as particles pass through the ureter.

A large number of Indian medicinal plants are being routinely used by practitioners of Ayurvedic system of medicine in the treatment of urinary stone disease[6]. Many plants have also been reported all over the world which are able to inhibit kidney stones[7]. Interestingly, the consumption of some of these plant products is also very high in areas where the incidence of the disease is reported to be very low. *Terminalia arjuna*, family: Combretaceae, whose medicinal value is well documented in the ayurvedic system, is an evergreen large decidous tree. The plant has been reported in ayurvedic system of medicine for derangement of all the three humours, *kafa, pitta* and *vayu*, and all sorts of conditions of cardiac failure[8], dropsy, antinfective[9], antiasthamatic, treatment of rheumatoid arthritis and is traditionally used to prevent kidney stone formation. Studies have also been conducting on *Terminalia arjuna* in support of its diuretic properties[10]. Aqueous extract of *Terminalia arjuna* bark is shown to protect the liver and kidney tissues against CCl_4_-induced oxidative stress probably by increasing antioxidative defense activities. Its aqueous extract prevents carbon tetrachloride induced hepatic and renal disorders[11].

Keeping in mind the complications arising due to the surgical treatment of kidney stones and great medicinal value of *Terminalia arjuna* owing to high content of polyphenols, flavonoids, antiurolithiatic properties of *Terminalia arjuna* bark have been studied using *in vitro* methods. Thus, the present study is aimed at investigating the antiurolithiatic effect of various fractions obtained after successive solvent extraction of *Terminalia arjuna* bark preceded by preliminary studies on its crude aqueous extract.

## MATERIALS AND METHODS

### Preparation of aqueous extract of *Terminalia arjuna*:

The dried bark of *Terminalia arjuna* was obtained from Natural Remedies Pvt. Ltd., Bangalore, India. A collection of voucher specimen is available with the company. The dried bark of *Terminalia arjuna* was boiled in distilled water. The extract was then filtered using Whatman No. 1 filter paper and the filtrate was evaporated *in vacuo* and dried using a rotary evaporator at 60°. The final dried sample was stored in labelled sterile bottles and kept at -20°. The final dried sample was reconstituted in water to 1000 μg/ml at the time of experiment and was referred to as aqueous extract of *Terminalia arjuna*.

### *In vitro* calcium phosphate (CaP) assay:

Effect of aqueous extract of *Terminalia arjuna* bark was studied on *in vitro* homogeneous system of initial mineral phase formation for CaP, its subsequent growth and demineralization by employing 5.0 ml system which was prepared by adding 0.5 ml of KH_2_PO_4_ (50 mM), 0.5 ml of CaCl_2_ (50 mM), 2.5 ml of Tris buffer (210 mM NaCl + 0.1 mM tris HCl) and increasing volume of the aqueous extract ranging from 0.2 ml to 1.5 ml by subsequently decreasing the volume of water ranging from 1.5 ml to 0.0 ml. This system was centrifuged at 4500 rpm and precipitates so obtained were dissolved in 5 ml of 0.1 N HCl. This 5 ml system for mineralization, already standardized in our laboratory was used to study the extent of *in vitro* mineral phase formation in the absence of any matrix[12,13]. For the growth, firstly 5 ml systems were prepared using standard protocols then again 5 ml systems were re-grown on the same tubes with the additions of increasing volumes of the extract. Calcium and phosphate were then estimated on the precipitates obtained and dissolved in 0.1 N HCl. In case of control no extract was added. To check the demineralization, again 5 ml system was prepared having no extract added to that and precipitates were obtained. To these precipitates, 2.5 ml of Tris buffer (210 mM NaCl+0.1 mM Tris HCl) and increased volumes of extract ranging from 0.2 ml to 1.5 ml with subsequently reduced volume of water was added and then centrifuged at 4500 rpm for 15 min. Calcium and phosphate were then estimated in supernatant obtained after centrifugation. The Ca^2+^ and HPO_4_^2-^ ions were estimated by the methods of Trinder[14] and Gomori[15], respectively. Percent inhibition of mineral phase in the presence of test plant aqueous extract was calculated as: % inhibition=((C—T)/C)×100, where T is the concentration of Ca^2+^ or HPO_4_^2-^ ion of the precipitate formed in test having plant aqueous extract ranging from 0.2 ml to 1.5 ml in the assay system and C is the concentration of Ca^2+^ or HPO_4_^2-^ ion of the precipitate formed in control system which had distilled water (Millipore) and no extract.

### *In vitro* calcium oxalate crystal growth assay:

Inhibitory activity of *Terminalia arjuna* extract was also checked on calcium oxalate crystal growth. A 4 ml system was prepared to check the effect of the extract in inhibiting growth of calcium oxalate crystals. In this system, 1 ml each of 4 mM calcium chloride and 4 mM sodium oxalate were added to a 1.5 ml of solution, containing NaCl (90 mM) buffered with Tris HCl (10 mM) pH 7.2. To this 30 μl of calcium oxalate monohydrate (COM) crystal slurry (1.5 mg/ml acetate buffer) was added. Consumption of oxalate begins immediately after COM slurry addition and was monitored for 600 sec by disappearance of absorbance at 214 nm[16]. When *Terminalia arjuna* extract is added into this solution, depletion of free oxalate ions will decrease if *Terminalia arjuna* extract inhibits calcium oxalate crystal growth. Rate of reduction of free oxalate was calculated using the baseline value and the value after 30 sec incubation with or without the extract. The relative inhibitory activity was calculated as follows: % relative inhibitory activity= ((C—S)/C)×100, where C is the rate of reduction of free oxalate without any extract and S is the rate of reduction of free oxalate with *Terminalia arjuna* extract.

### Bioactivity guided successive solvent extraction:

To isolate the phytochemicals from the bark of *Terminalia arjuna*, successive solvent extraction method was used[17]. This method employs treatment of various organic solvents to isolate phytochemicals of the same polarity as that of the organic solvent in which they are dissolved. Process of extraction was started with 300 g of dried powder of the bark of the plant soaked in 6 liters of ethanol (99.9%) for 24 h. The extract was filtered through muslin cloth and was subjected to rotary evaporator at 65° for 24 h to get a viscous liquid. This liquid was put in petriplates at 40° overnight. The dried extract so obtained was extracted with 150 ml of hexane, dichloromethane, ethyl acetate and n-butanol for 24 h in separating funnel successively. All the extracts were then dried in rotary evaporator at 40° separately. Various qualitative tests were then performed on all the dried fractions obtained after successive solvent extraction. Presence of tannins was confirmed by performing the ferric chloride test. For this, 0.5 g of all extracts was boiled with 20 ml of double distilled water and filtered. A few drops 1% ferric chloride were added and observed for brownish green coloration. The presence of saponins and terpenoids was determined by using 1 g of all extracts boiled in 10 ml of double distilled water. Formation of froth on vigorous shaking and mixing of this froth with 3 drops of olive oil showing formation of an emulsion confirmed presence of saponins. To test the presence of flavonoids, 5 ml of diluted ammonia was added to all fractions mixed in double distilled water (10%) followed by addition of few drops of concentrated sulphuric acid. A yellow color formation indicated presence of flavonoids. This yellow color disappeared on standing. However, for the presence of terpenoids, Salkowski test was performed[18]. Five milliliters of each extract was mixed in 2 ml of chloroform and 3 ml of conc sulphuric acid was carefully added to form a layer. Formation of reddish brown coloration at the interface confirmed the presence of terpenoids. The presence of total phenolic compounds of *arjuna* were expected in more polar solvents like ethyl acetate and butanol. Hexane and dichloromethane extracts were dissolved in 5 ml DMSO whereas ethyl acetate and butanol extracts were dissolved in 5 ml of double distilled water separately. Inhibitory potential of each fraction thus obtained was then checked on CaP assay and COM crystal growth *in vitro*.

### Qualitative test for the confirmation of saponins in butanol fraction by TLC:

TLC was performed on 20×20 cm silica gel plates (0.25 mm silica gel). To this plate 15 μl of sample was loaded. The solvent system used was butanol:water:acetic acid, 12:2:1 (lower phase). Plates were sprayed with *p*-anisaldehyde:acetic acid:sulfuric acid (1:2:100) and heated for 10 min at 110° to visualize saponins appeared in the form of blue bands[19].

## RESULTS AND DISCUSSION

**Fig. 1:**
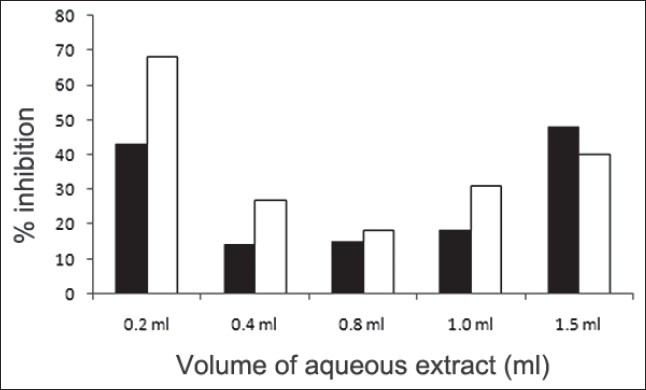
Effect of *Terminalia arjuna* extract on initial mineral phase formation of calcium phosphate (CaP) ions Percent inhibition of calcium (■) and phosphate (□) ions precipitation by aqueous extract of *Terminalia arjuna* in initial mineral phase formation. Volumes used of aqueous extract were 0.2, 0.4, 0.8, 1.0 and 1.5 ml. Maximum calcium ion inhibition of 48% was seen with 1.5 ml followed by 0.2, 1.0, 0.8 and 0.4 ml. However, for phosphate ions, maximum inhibition was found to be 68% with 0.2 ml followed by 1.5, 1.0, 0.4 and 0.8 ml

**Fig. 2:**
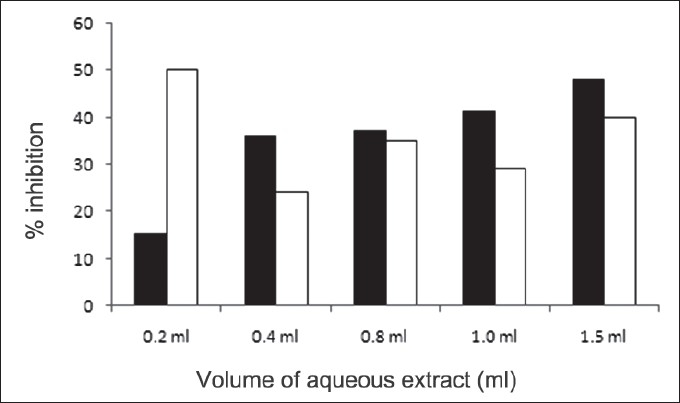
Effect of *Terminalia arjuna* extract on growth of preformed mineral phase of calcium phosphate (CaP) ions Percentage inhibition of calcium (■) and phosphate (□) ions precipitation by aqueous extract of *Terminalia arjuna* in case of growth of preformed initial mineral phase formation. Here, maximum inhibition of calcium ion was at 1.5 ml (48%) followed by 1.0 (41%), 0.8 (37%), 0.4 (36%) and 0.2 ml (15%). Inhibition of phosphate ion was found to be maximum with 0.2 ml (50%) of extract followed by 1.540%), 0.8 (35%), 1.0 (29%) and 0.4 ml (24%).

**Fig. 3:**
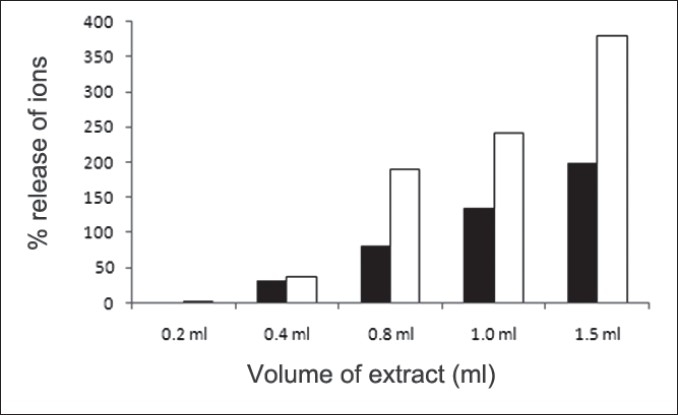
Effect of *Terminalia arjuna* extract on demineralization of calcium phosphate (CaP) ions Effect of aqueous extract of *Terminalia arjuna* on demineralization of calcium (■) and phosphate (□) ions in terms of percentage release of ions. In case of calcium maximum release was observed with 1.5 ml of the extract (197%). Release of calcium ions increased with the increase in the volume of the extract. Minimum calcium release was seen with 0.2 ml of aqueous extract which was 0%. In case of phosphate ions, again maximum release was observed with 1.5 ml of extract (380%) followed by 1.0 (243%), 0.8 (190%), 0.4 (38%) and least with 0.2 ml (2%).

**Fig. 4:**
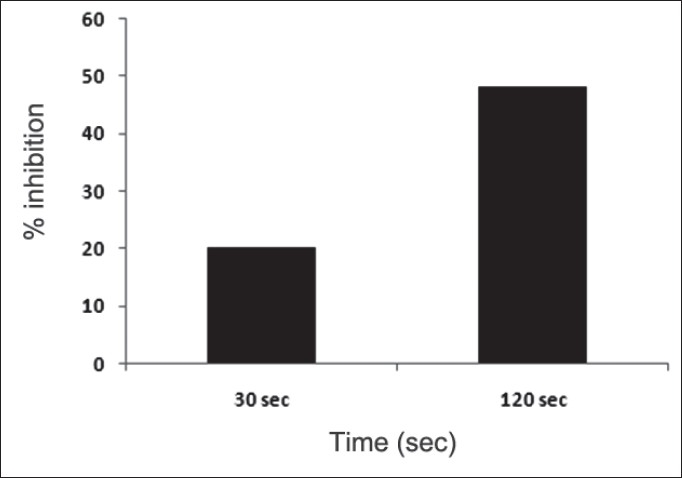
% inhibition of COM crystal growth by *Terminalia arjuna* extract Percentage inhibition of 20 % was found at 30 sec and 48% at 120 sec time interval when 15 μl of aqueous extract of *Terminalia arjuna* was used against growth of calcium oxalate monohydrate (COM) crystals.

**Fig. 5:**
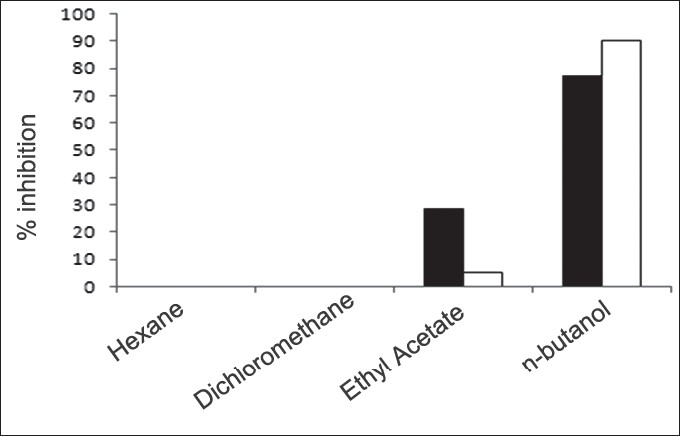
Inhibitory effect of *Terminalia arjuna* extract fractions on the initial mineral phase formation of calcium phosphate (CaP) ions Inhibitory activity of fractions obtained after performing successive solvent extraction against CaP initial mineral phase formation. Out of all the fractions obtained, hexane and dichloromethane fractions did not show any inhibition in calcium (■) and phosphate (□) ion precipitation. Ethyl acetate fraction showed 28.26% inhibition of calcium and 5 % inhibition of phosphorus in initial mineral phase formation. However, n-butanol fraction (0.1 ml) showed enhanced inhibition, 77% for calcium ions and 90% for phosphate ions

**Fig. 6:**
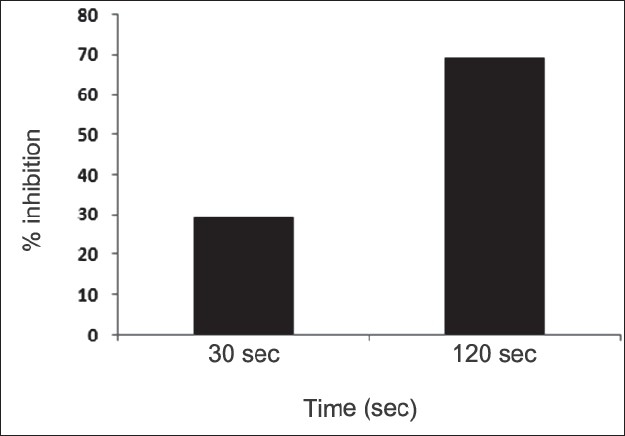
Effect of n-butanol fraction on COM crystal growth. n-butanol fraction (10 μl of 10 times diluted fraction) showed inhibition of 29% and 69% at time intervals of 30 and 120 sec, respectively of calcium oxalate monohydrate (COM) crystal growth

First, the studies were conducted by using aqueous extract on initial mineral phase formation of CaP, its subsequent growth and demineralization of initial mineral phase formation. In initial mineral phase formation, volumes used of aqueous extract were 0.2, 0.4, 0.8, 1.0 and 1.5 ml. It was found that in the case of *Teminalia arjuna* bark extract maximum calcium ion inhibition was at 1.5 ml followed by 0.2, 1.0, 0.8 and 0.4 ml. However, for phosphate ions, maximum inhibition was found to be at 0.2 ml followed by 1.5, 1.0, 0.4 and then 0.8 ml (fig. 1). After examining results of growth of preformed initial mineral phase, it was observed that maximum inhibition of calcium ion was at 1.5 ml followed by 1.0, 0.8, 0.4 and 0.2 ml. Inhibition of phosphate ion was found to be maximum with 0.2 ml of extract followed by 1.5, 0.8, 1.0 and 0.4 ml (fig. 2). Finally, demineralization was conducted using 0.2, 0.4, 0.8, 1.0 and 1.5 ml of extract and it was observed that maximum calcium and phosphate ions released were at 1.5 ml followed by 1.0, 0.8, 0.4, 0.2 ml (fig. 3). After CaP, inhibitory activity of the crude aqueous extract was checked on COM crystal growth and it again was fond to be inhibitory. A percentage inhibition of 20% was found at 30 sec and 48% at 120 sec time interval (fig. 4). After checking for the inhibitory activity, isolation of various phytochemicals was done from dried powder of *Terminalia arjuna* by bioactivity guided successive solvent extraction method. Again inhibitory activity of fractions obtained was checked on CaP initial mineral phase formation and on COM crystal growth. Out of all the fractions obtained, (hexane, dichloromethane, ethyl acetate and n-butanol) hexane and dichloromethane fractions did not show any inhibition in any of the stones formation. However, in case of ethyl acetate, it showed a calcium inhibition of 28.26% and phosphate inhibition of 5% in initial mineral phase formation of CaP by using 0.1 ml of extract. Inhibitory activity for COM crystal growth was nil in ethyl acetate fraction. Out of all the fractions obtained, n-butanol fraction dissolved in double distilled water showed maximum inhibition both in case of CaP initial mineral phase formation and COM crystal growth. A percentage inhibition of 77% for calcium and 90% for phosphate was reported during initial mineral phase formation of CaP with 0.1 ml of n-butanol fraction (fig. 5). In case of COM crystal growth inhibition was shown by only n-butanol fraction. It came out to be 29% at 30 sec and 69% at 120 sec when sample was diluted 10 times and 10 μl of diluted sample was loaded (fig. 6). Separated samples were then checked for the presence of various phytochemicals and presence of good amount of saponins, tannins and traces of terpanoids were observed in the most potent n-butanol fraction. Presence of saponins in n-butanol fraction was further confirmed by performing thin layer chromatography (TLC). Blue colored band was observed after spraying with *p*-anisaldehyde:acetic acid:sulfuric acid (1:2:100).

Here, in the present work done, we have studied detailed behavior of phytochemicals present in the bark of *Terminalia arjuna* in terms of their antiurolithiatic nature. Under normal conditions, biomineralization, like the formation of bone and teeth, takes place in controlled biological situations. Uncontrolled pathological crystallization occurs when solvent becomes supersaturated leading to the formation of precipitates in the body called as kidney stones[20]. This is a condition when ionic product becomes greater than solubility product and hence precipitation of ions takes place. Many factors contribute to the formation of kidney stones[21]. Kidney stones often occur when urine becomes too concentrated[22]. This causes calcium, oxalate and phosphate or other chemicals in the urine to form crystals on the inner surfaces of kidneys. This stage is called as initial mineral phase formation. Over the time these crystals may combine to form a small, hard mass called as stones and stage is referred as subsequent growth of crystals. Retardation of such growth of calcium oxalate monohydrate stones by *Tamarindus indica* pulp was also observed in the studies carried by our group[23].

Besides this, an uneven balance of acid in the urine and lack of substances in the urine that prevent the growth of crystals also affect the ability of stone forming substances to remain dissolved. Ayurvedic system plays a vital role in providing plant based medicines to overcome this disease. Recently, studies in our lab have also been done on medicinal plants like Trachyspermum ammi showing its antiurolithiatic properties[24]. Here, in the present study, antiurolithiatic properties of *Terminalia arjuna* have been revealed. Preliminary studies on crude aqueous extract showed that *Terminalia arjuna* is capable of inhibiting formation of CaP and COM crystals. For checking inhibitory activity on COM crystal growth, it was seen that inhibition increases with increase in time, probably the secondary metabolites present in *Terminalia arjuna* extract interfere with the growth of calcium and oxalate onto the slurry and thus inhibits the growth process. *Terminalia arjuna* is well known for its high phenolic content which after performing successive solvent extraction comes in n-butanol fraction. Successive solvent extraction method was used to fractionate the phytochemicals from dried extract obtained after ethanolic washing of crude extract. Here, phytochemicals were separated on the basis of their polarity. First, non polar organic solvent was added to the separating funnel containing dried powder obtained after drying ethanolic extract of crude powder in rotary evaporator for 24 h. Non polar molecules like lipids, got dissolved in non polar solvent like hexane. Similarly, alkaloids, tannins and saponins got dissolved in solvents having same polarity as they are having. In this way, the most polar molecules remained in the last and polar solvents like butanol was used to dissolve them[15]. After performing froth test for the presence of saponins in butanol fraction, TLC showed the blue colored band confirming the presence of saponins. Saponin rich fractions of other plants like, Herniaria hirsute[25] has also been found to be a great inhibitor of calcium stone formation not only *in vitro* but in vivo too. It has also been reported in the literature that casuarinin extracted from *Terminalia arjuna* attenuates H_2_O_2_-induced oxidative stress, decreases DNA oxidative damage and prevents the depletion of intracellular GSH in MDCK cells[26].

Thus, after conducting a series of experiments it was observed that butanol fraction containing high amount of saponins was able to inhibit initial mineral phase formation of CaP and growth of COM crystals along with the crude aqueous extract of *Terminalia arjuna*. It was quite evident from the studies conducted that inhibition was enhanced when checked with the purified butanol fraction.

Hence, it is concluded that saponins present in the purified n-butanol fraction from the bark of *Terminalia arjuna* are able to inhibit CaP mineralization and COM crystal growth *in vitro*. Thus, this study puts forth the possibility of using phytochemicals present in the plants as therapeutic agents to treat urolithiasis, but still it warrants further investigation both, in elaborated experimentation and human trials.
